# Supplementary material for: Mono-Rhamnolipid Biosurfactants Synthesized by Pseudomonas aeruginosa Detrimentally Affect Colorectal Cancer Cells
Source: Pharmaceutics. 2022 Dec 14;14(12):2799. doi: 10.3390/pharmaceutics14122799 (PMC9782318; doi:10.3390/pharmaceutics14122799)
Supplement: Supplementary file 1 [file pharmaceutics-14-02799-s001.zip › pharmaceutics-2024987-supplementary.pdf]

Supplementary Materials: Mono-Rhamnolipid Biosurfactants Synthesized by *Pseudomonas aeruginosa* Detrimentally Effect Colorectal Cancer Cells

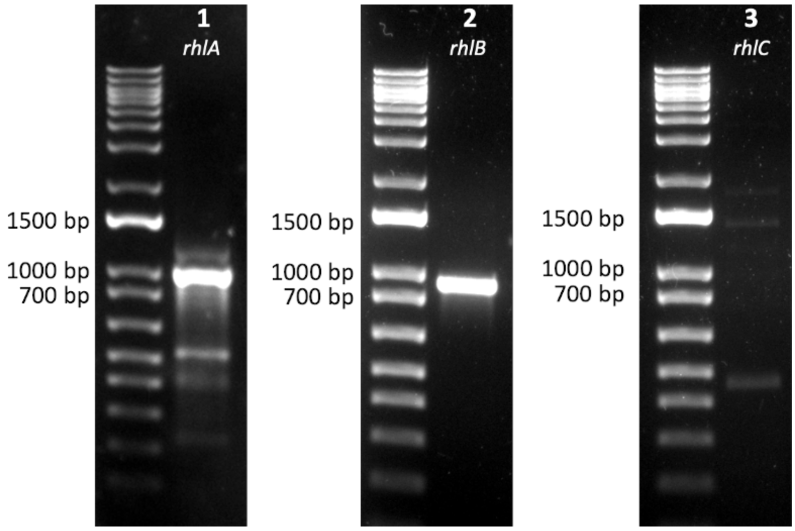

Figure S1. Amplified DNA fragments resulting from the PCR screening for RL biosynthesis genes *rhlA*, *rhlB* and *rhlC* in *P. aeruginosa* PAO1 Δ*rhlC*. The lack of an amplification product at ≈ 800 bp for *rhlC* is confirmation of mutation of this gene within the strain.

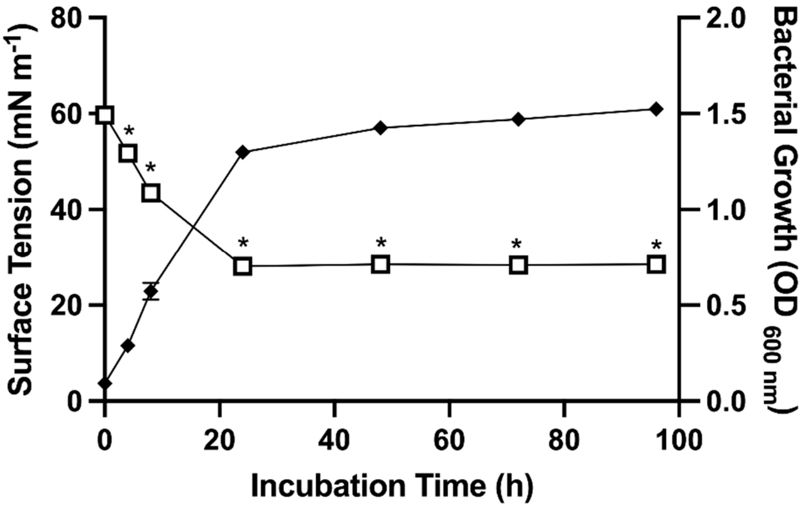

Figure S2. Growth and surface tension measurements of *P. aeruginosa* PAO1 Δ*rhlC* over 96 h. Bacterial growth of in NB supplemented with 1% (*v*/*v*) glycerol (◆). Surface tension measurement of cell-free supernatant samples obtained from cultures over time (□). Error bars represent standard deviation from the mean ($n = 3$). Analysis of surface tension measurements was by two-way ANOVA with post hoc. comparison to surface tension at t = 0 h ($* = p \leq 0.05$).

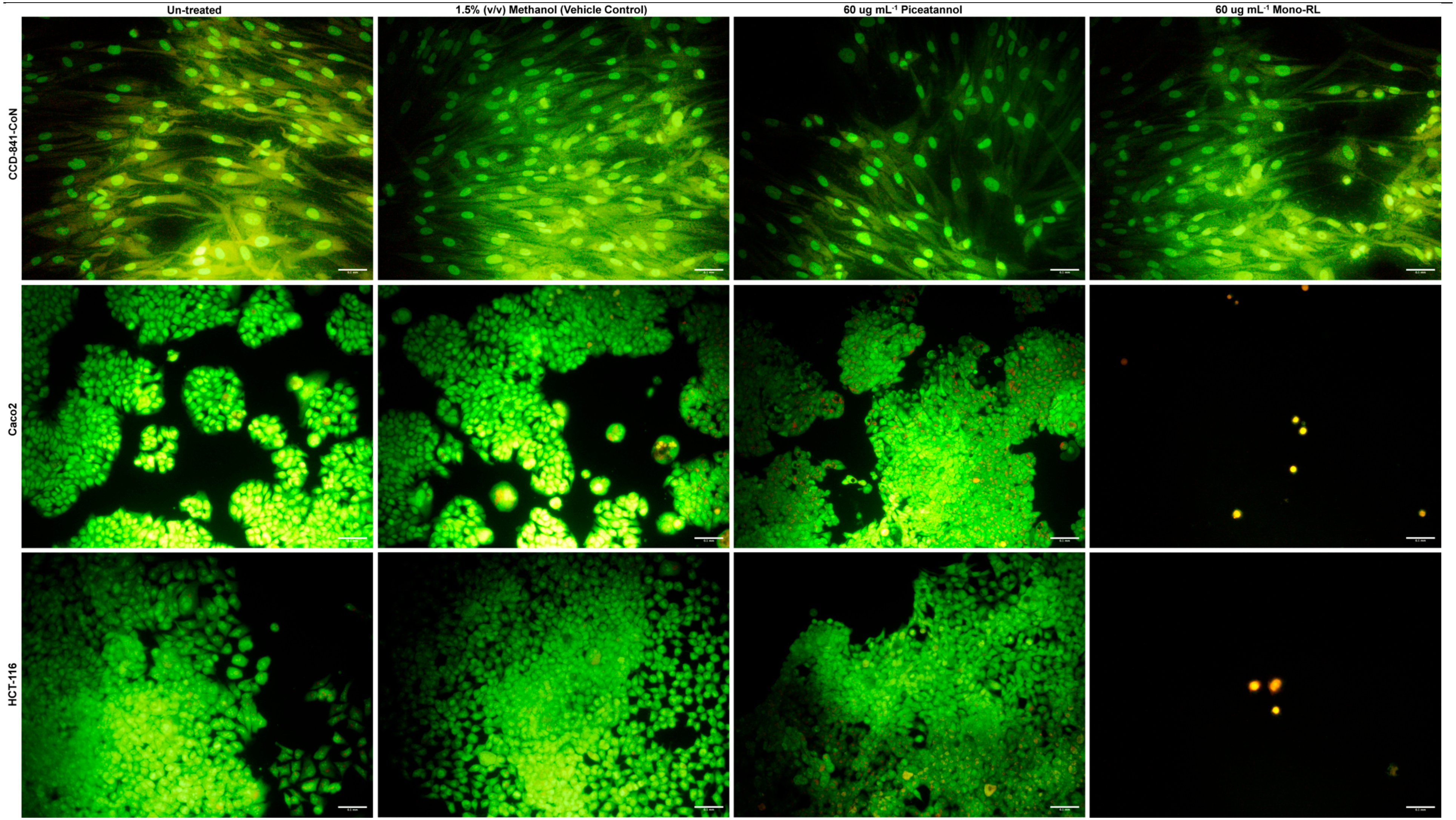

Figure S3. Images of AO/ PI-stained cell lines. CCD-841-CoN, Caco2 and HCT-116 either un-treated or following 24 h of treatment with 1.5% (*v*/*v*) methanol (vehicle control); 60 ug mL-1 piceatannol; or 60 ug mL-1 mono-RL. Cells were imaged at 200× magnification, scale bar = 100 µm.

Table S1. PCR primer pairs used in this study and their melting temperatures. Oligonucleotide primers were obtained in lyophilized form from *Eurofins Genomic*s, Germany, reconstituted in nuclease free distilled H_2O at a concentration of 100 µM and utilized in each PCR at a working concentration of 0.5 µM.

Gene Amplified	Primer Pair	Sequence (5′-3′)	Melting Temp. (°C)
16S rRNA	27-F	AGAGTTTGATCMTGGCTCAG	56.3
	1492-R	CGGTTACCTTGTTACGACTT	55.3
	907-R *	CCGTCAATTCMTTTRAGTTT	51.1
rhlA	*rhlA*-F	AGACGTACTCGTAGACCGGC	61.4
	rhlA-R	AAGGACGACGAGGTGGAAAT	57.3
rhlB	*rhlB*-F	CCCGTAGTTCTGCATCTGGT	59.3
	rhlB-R	AACTGCAACGCTTTCTCGAT	55.3
rhlC	*rhlC*-F	GTCCACGTGGTCGATGAAC	58.8
	rhlC-R	CCTGGTCGACTCGTCATTCT	59.3

* Primer 907-R used as internal primer for 16S rRNA sequencing
